# Depressive Symptoms among Bariatric Surgery Candidates: Associations with Stigmatization and Weight and Shape Concern

**DOI:** 10.3390/nu16040510

**Published:** 2024-02-12

**Authors:** Alexandra Fabrig, Ricarda Schmidt, Thomas Mansfeld, Johannes Sander, Florian Seyfried, Stefan Kaiser, Christine Stroh, Arne Dietrich, Anja Hilbert

**Affiliations:** 1 Behavioral Medicine Research Unit, Department of Psychosomatic Medicine and Psychotherapy, Integrated Research and Treatment Center AdiposityDiseases, University of Leipzig Medical Center, Stephanstrasse 9a, 04103 Leipzig, Germany; alexandra.fabrig@medizin.uni-leipzig.de (A.F.);; 2 Department of General Surgery, Asklepios Clinic, 22559 Hamburg, Germany; 3 Schön Klinik Hamburg Eilbek, Obesity Clinic, 22081 Hamburg, Germany; 4 Department of General, Visceral, Transplant, Vascular and Pediatric Surgery, University Hospital, University of Würzburg, 97080 Würzburg, Germany; 5 Department of Visceral, Pediatric and Vascular Surgery, Hospital Konstanz, 78464 Konstanz, Germany; 6 Department of Surgery, SRH Wald-Klinikum, Adipositas Zentrum, 07548 Gera, Germany; 7 Department of Surgery, Clinic for Visceral, Transplantation, Thoracic and Vascular Surgery, University Hospital Leipzig, 04103 Leipzig, Germany

**Keywords:** bariatric surgery candidates, weight bias internalization, weight-related experienced stigmatization, depressive symptoms, weight and shape concern

## Abstract

Bariatric surgery candidates (BSC) are a highly vulnerable group for mental health impairments. According to the theoretical model of weight stigma, weight-related experienced stigmatization (ES) negatively influences mental health through weight bias internalization (WBI). This study tested this model among BSC and investigated whether this association depends on a negative body image in terms of weight and shape concern as a potential moderator. As part of a German multicenter study, ES, WBI, weight and shape concern, and depressive symptoms were assessed via self-report questionnaires among *n* = 854 BSC. Simple and moderated mediation analyses were applied to analyze whether WBI influences the relationship between ES and depressive symptoms, and whether this influence depends on weight and shape concern. WBI significantly mediated the relationship between ES and depressive symptoms by partially reducing the association of ES with depressive symptoms. Weight and shape concern emerged as significant moderators in the overall model and specifically for associations between WBI and depressive symptoms. The results suggest that the association between ES and depressive symptoms among BSC is stronger in those with high WBI. This association is strengthened by weight and shape concern, especially at low and mean levels. Studies evaluating longitudinal associations between weight-related stigmatization and mental health are indicated, as well as intervention studies targeting WBI in order to reduce adverse effects of ES on mental health in BSC.

## 1. Introduction

The worldwide prevalence of obesity is increasing [1,2,3], with 13% of adults exceeding a body mass index (BMI; kg/m^2^) of 30 kg/m^2^ according to the World Health Organization [4]. In 2014, 2.3% of men and 5.0% of women globally met the criteria for obesity class 2 (35.0 kg/m^2^ ≤ BMI < 40.0 kg/m^2^) and 3 (BMI ≥ 40.0 kg/m^2^) [3]. Due to associations with physical [5] and mental health impairments including depressive symptoms [6], obesity poses a major economic challenge to healthcare systems [7]. The standard treatment of obesity, behavioral weight loss treatment, including nutritional, physical activity, and behavioral, shows overall small effects on health outcomes [8]. Although, the adjunct of the subcutaneous application of semaglutide can optimize the effects [9], bariatric surgery is the most efficacious treatment for patients with severe obesity, including obesity class 2 with physical comorbidity and class 3 leading to significant weight loss of 20–35% and long-term improvements in physical and mental comorbidities [10,11].

Among individuals with obesity, bariatric surgery candidates (BSC) comprise a notably vulnerable group to mental health impairments, given that up to 58% of these patients present with a mental disorder, especially affective and anxiety disorders, as well as eating disorders [12]. Rates of depression are significantly higher in BSC than in those seeking behavioral weight loss treatment [13]. However, so far, mechanisms related to the development of psychopathology such as depressive symptoms among BSC are not fully understood. Based on Tylka et al.’s [14] theoretical model of weight stigma, which is based on both longitudinal and cross-sectional data and supported by recent cross-sectional research [15,16,17,18], experienced stigmatization (ES) may lead to weight bias internalization (WBI) and/or body shame, thereby negatively influencing psychological well-being. While ES describes negative experiences related to one’s weight [19], with weight-based teasing being the most common type of ES [20], WBI denotes individual beliefs that negative stereotypes related to one’s weight are true for oneself. A variety of cross-sectional studies demonstrated that both ES and WBI increase with higher BMI [8,20,21,22], and are negatively associated with mental health including depressive symptoms [23,24,25,26,27,28,29]. This pattern is especially pronounced in BSC compared to individuals with obesity undergoing behavioral weight loss treatment [30,31], with prospective experimental evidence highlighting stronger negative effects on mental health for WBI than for ES [32]. ES and WBI were found to be associated with medium effect size, according to a systematic review with predominantly cross-sectional evidence, in adults from the population [27], and based on a cross-sectional study in BSC [21]. Notably, a systematic review revealed that ES in terms of weight-based teasing in childhood was longitudinally and cross-sectionally positively associated with depressive symptoms in both childhood and adulthood [33]. Another recent cross-sectional study among treatment-seeking adults with obesity who have experienced and internalized weight stigma found a high percentage of depressive symptoms [29].

Supporting Tylka et al.’s [14] model, a recent systematic review demonstrated that WBI may function as a mediator between ES and psychological well-being, including depression, disordered eating, and body dissatisfaction, in community-based and clinical populations [34]. Specifically, in adult patients with obesity participating in a behavioral weight loss program, ES had a direct and an indirect effect on depression through WBI [35]. Among BSC, cross-sectional evidence identified the interplay of WBI, body shame, and internalized shame as mediators in the relationship between ES and depression, though WBI as a single mediator was not significant [36]. Body shame has been highly associated with eating disorder symptoms, including weight and shape concern [37,38,39], and depressive symptoms in BSC. At the same time, weight and shape concern and WBI were cross-sectionally highly associated in BSC [31]. However, nothing is known about the potential impact of weight and shape concern on the relationship between ES, WBI, and mental health among BSC, specifically whether weight and shape concern strengthen the effect of WBI on the association between ES and depressive symptoms.

In this context, the aim of this cross-sectional study was to investigate, first, the mediating role of WBI on the relationship between ES and depressive symptoms among BSC and, second, the potential influence of weight and shape concern on the association between ES, WBI, and depressive symptoms. Based on Tylka et al.’s [14] theoretical model of weight stigma and related evidence, it was hypothesized that ES directly and indirectly (through WBI) will be related to depressive symptoms, and that this mediation will be moderated by BSC’s weight and shape concern. An investigation of these associations was deemed to be of high clinical relevance, since both WBI and weight and shape concern may serve as potential intervention targets to improve BSC’s mental health.

## 2. Materials and Methods

### 2.1. Sample

This study is part of the multicenter Psychosocial Registry for Bariatric Surgery (PRAC) study, which longitudinally assesses psychosocial aspects in a consecutive sample of patients seeking bariatric surgery in six participating study centers in Germany. Inclusion criteria for the PRAC study were a minimum age of 18 years and a planned bariatric surgery. Patients were excluded due to insufficient German language skills and inability to comply with the study protocol. Based on an eligible sample of *n* = 978, data on the self-reported measures of interest (see below) were missing for *n* = 124, leaving a total sample of *n* = 854 adult BSC, recruited between March 2012 and March 2023. All patients provided written informed consent before study participation. Data collection proceeded independently of clinical treatment, and all patients were informed that study data would be treated as strictly confidential and inaccessible to the surgical team.

### 2.2. Measures

This study used PRAC baseline data from well-established self-report questionnaires on weight and shape concern, weight-related stigmatization, and depressive symptoms, assessed prior to bariatric surgery.

#### 2.2.1. Predictor Variable: Experienced Stigmatization

The German version of the 6-item Perception of Teasing Scale (POTS [40,41]) was used to assess how often participants had been the target of weight stigmatization by others in their childhood on a 5-point scale ranging from 0 (“never”) to 4 (“very often”). The effect of teasing on the individuals, a second subscale of the POTS, was not evaluated in this study. All responses were summed up to a total score, with higher scores representing more frequently perceived teasing (Cronbach’s alpha in the present study α = 0.97).

#### 2.2.2. Outcome Variable: Depressive Symptoms

The 9-item subscale of the German version of the Patient Health Questionnaire (PHQ-D [42,43,44]) was used to screen for depressive symptoms based on the Diagnostic and Statistical Manual of Mental Disorders (DSM-IV [45]). All items were rated on a 4-point Likert scale ranging from 0 (“not at all”) to 3 (“nearly every day”), with higher sum scores indicating higher severity of depression (α = 0.85).

#### 2.2.3. Mediator Variable: Weight Bias Internalization

The German version of the 11-item Weight Bias Internalization Scale (WBIS [46,47]) was used to assess the level of weight bias internalization describing someone’s belief that negative stereotypes and negative self-statements about persons with overweight or obesity apply to him- or herself. From April 2015, the WBIS was replaced by the German version of the Modified Weight Bias Internalization Scale (WBIS-M [47,48]), which assesses WBI across various weight statuses. Although the WBIS-M showed slightly better psychometric properties than the WBIS, both measures showed acceptable internal consistency as well as convergent and divergent validity [49]. In favor of good readability, only “WBIS” is referred to in tables and figures. All items were rated on a 7-point Likert scale ranging from 1 (“strongly disagree”) to 7 (“strongly agree”). According to the results of psychometric analyses, item 1 was removed before computing the mean score [47]. A higher mean score indicates greater internalized weight stigma (α = 0.87).

#### 2.2.4. Moderator Variable: Weight and Shape Concern

The 5- and 8-item subscales of the German Eating Disorder Examination Questionnaire (EDE-Q [50,51]) on weight concern and shape concern were combined to measure a composite covering both weight and shape concern [52,53]. The items were rated on a 7-point Likert scale ranging from 0 (“no day”/“not at all”) to 6 (“everyday”/“extremely”) with higher mean scores indicating greater weight and shape concern (α = 0.84).

#### 2.2.5. Control Variables

Sociodemographic characteristics were assessed by self-report, including participants’ age, sex (male, female), and education (≥10 school years, <10 school years). BMI was calculated from participants’ measured weight and height using calibrated scales.

### 2.3. Data Analysis

**Figure 1:**
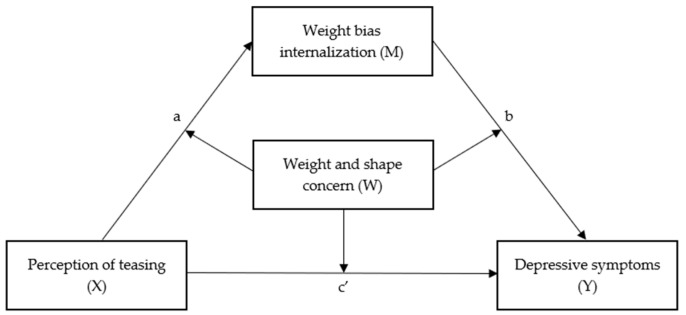
Moderated mediation model.

A priori power analysis was calculated to determine the minimum sample size for detecting medium-sized effects with a statistical power of 0.80. For mediation analyses (small-sized a path, medium-sized b path, see Figure 1; percentile bootstrapping), *n* = 406 patients were required [54]. All statistical analyses were performed using IBM SPSS Statistics Version 29 and a two-tailed significance level of α = 0.05. Prior to conducting the main analyses, all variables were screened for plausibility and outliers. Pearson and Spearman correlation analyses were run to examine bivariate associations between all study variables and to identify relevant sociodemographic control variables.

In order to examine whether there was an indirect effect of ES on depressive symptoms through WBI, a simple mediation analysis was conducted using Model 4 from the SPSS PROCESS macro 4.0 [55]. Secondly, in order to investigate the moderating influence of weight and shape concern on all paths of the mediation model, a moderated mediation analysis (Process Model 59) was conducted (see Figure 1 for the hypothesized model). Both the mediation-only and moderated mediation models were controlled for sex, age, and BMI due to significant associations of these variables with model variables. Bootstrapping was applied, which involved repeated sampling from the dataset with replacement (i.e., 10,000 bootstrap resamples), in order to achieve an approximation of the sampling distribution of the indirect effect and to generate 95% confidence intervals for these effects. For illustrative purposes, the moderating effect of weight and shape concern was calculated at three different levels of the moderator (i.e., −1 *SD*, mean, +1 *SD*).

## 3. Results

### 3.1. Sample Characteristics

The sample had a mean age of 46.8 ± 11.6 years, with *n* = 547 (67.2%) women (Table 1). Mean BMI was 48.7 ± 8.0 kg/m^2^, with the majority of patients having obesity class III (*n* = 754, 88.3%). Most participants were married (*n* = 430, 51.7%) and had at least 10 years of education (*n* = 579, 76.3%).

Associations between all study variables can be found in Table 2. Among possible covariates, age, sex, and BMI, but not education, were significantly associated with the predictor, outcome, mediator, and moderator.

### 3.2. Mediation

**Figure 2:**
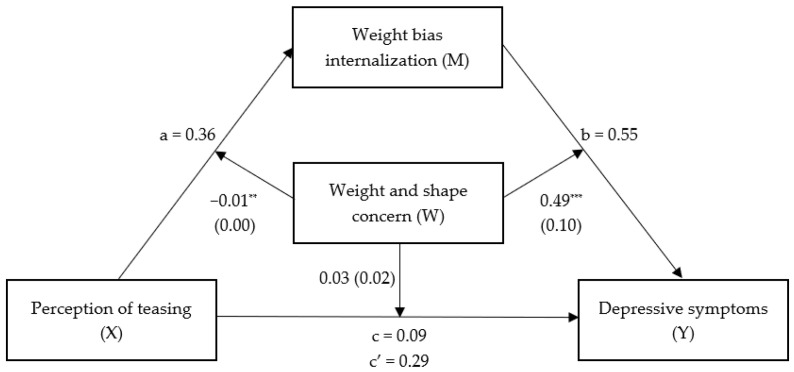
Moderated mediation model displayed with coefficients and standard errors. Mediation coefficients displayed are standardized. ** *p* < 0.01. *** *p* < 0.001.

The overall prediction by the model of greater depressive symptoms by more frequent ES through the indirect effect of WBI was significant (*F*(5, 848) = 89.331, *p* < 0.001), accounting for 35% of variance (see Table 3 and Figure 2). The inclusion of WBI in the model reduced the direct effect of ES on depressive symptoms significantly (*p* < 0.001). Thus, WBI was found to be a partial mediator of the association between ES and depressive symptoms.

### 3.3. Moderated Mediation

**Figure 3:**
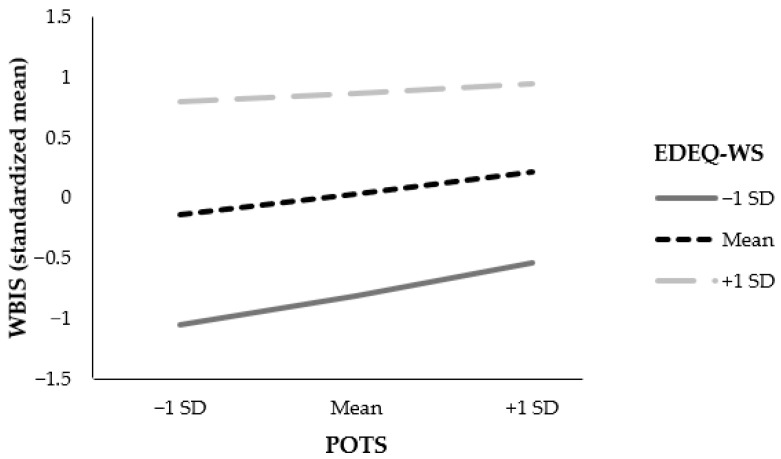
Conditional effect of perception of teasing on weight bias internalization at values of weight and shape concern. Note. POTS = Perception of Teasing Scale; WBIS = Weight Bias Internalization Scale; EDE-Q WS = Eating Disorder Examination Questionnaire: composite of weight and shape concern.

The overall prediction by the model of greater depressive symptoms by more frequent ES through WBI, while considering weight and shape concern, was significant (F(8, 845) = 74.140, *p* < 0.001), accounting for 41% of variance (see Table 4 and Figure 2). The interaction between the effects of ES and weight and shape concern on WBI was statistically significant, *p* = 0.001. Specifically, the conditional effect of ES on WBI was significant for low and mean values of weight and shape concern, both *p* < 0.001, while high values of weight and shape concern did not moderate the effect between ES and WBI, *p* = 0.063. The results thus indicate that the effect of ES on WBI was stronger for patients with low and mean weight and shape concern—see Figure 3.

**Figure 4:**
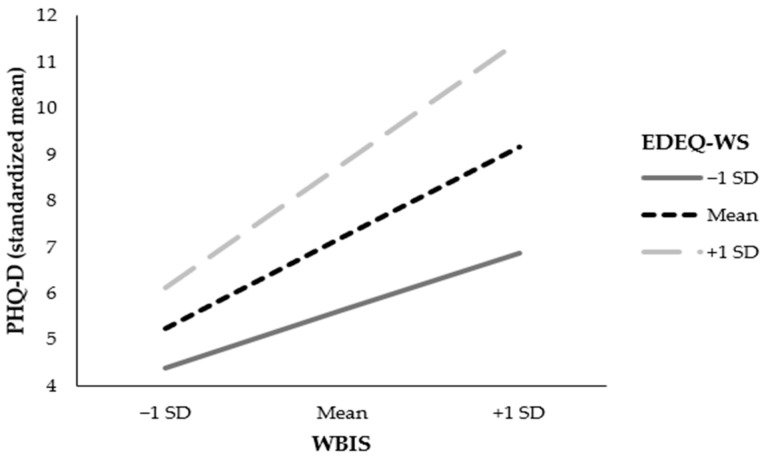
Conditional effect of weight bias internalization on depressive symptoms at values of weight and shape concern. Note. WBIS = Weight Bias Internalization Scale; PHQ-D = Patient Health Questionnaire Depression Scale; EDE-Q WS = Eating Disorder Examination Questionnaire: composite of weight and shape concern.

The moderating effect of weight and shape concern on the association between WBI and depressive symptoms was significant, *p* < 0.001. Specifically, the conditional effect of WBI on depressive symptoms was significant for low, mean, and high values of weight and shape concern, all *p* < 0.001, indicating that they increased the association between WBI and depressive symptoms—see Figure 4.

There was no moderating effect of weight and shape concern on the association between ES and depressive symptoms, *p* = 0.164, indicating that weight and shape concern did not strengthen or weaken the respective association.

The moderation of the indirect effect of ES on depressive symptoms through WBI was significant for low and mean values, but not for high values of weight and shape concern, indicating that the indirect effect of ES on depressive symptoms through WBI was stronger for patients with low and mean weight and shape concern.

## 4. Discussion

This cross-sectional study was the first to test the theoretical model of weight stigma [14], specifying associations between experienced stigmatization (ES), weight bias internalization (WBI), and depressive symptoms among bariatric surgery candidates (BSC), adding weight and shape concern as a potential moderator. In a large baseline sample of BSC, we found mediating effects of WBI on the relation between ES and depressive symptoms, and weight and shape concern moderated this mediation.

Notably, compared to a community sample of adults with overweight or obesity [18], the association between ES and WBI was smaller in this study of BSC, but similar to another recent study in BSC [21]. Supporting the suggested model and prior population-based research [34], WBI was here found to mediate the association between ES and depressive symptoms. The result is also in line with cross-sectional findings in individuals opting for surgical and non-surgical intervention with obesity [35], showing an indirect effect of more ES on lower mood through WBI. A recent cross-sectional study in BSC did not find a significant separate effect of WBI on ES and depressive symptoms, but only in combination with high internalized shame and body shame, and low self-compassion [36]. Based on the present results, depressive symptoms among BSC, who experienced frequent weight-based teasing in childhood, were stronger in those with a higher than lower internalized weight bias. Thus, health care professionals might pay particular attention to patients undergoing bariatric surgery, believing that negative stereotypes about weight apply to themselves, in order to improve their psychological well-being in relation to ES in childhood.

Based on Tylka et al.’s model [14], there may be a moderation effect caused by body shame on associations between ES and mental health. The present study extended this model by testing the moderating effect of weight and shape concern. As hypothesized, a significant moderating effect of weight and shape concern was found: weight and shape concern strengthened the mediating effect of WBI on the association between ES and depressive symptoms among BSC, especially in those with low and mean levels of weight and shape concern. To understand this result, it is important to evaluate the separate path connections. Weight and shape concern at all levels had a large moderating effect on the relation between WBI and depressive symptoms. This result goes in line with findings from a cluster analysis revealing that individuals with overweight or obesity and weight concern showed low levels of happiness and positivity [56]. Similarly, a cross-sectional study demonstrated that WBI was highly correlated with depressive symptoms and weight and shape concern among BSC [57]. With a significant but small effect, the composite of weight and shape concern moderated the relation between ES and WBI, indicating that the lower the patients’ weight and shape concern, the stronger the positive relation between ES and WBI. Experienced teasing in childhood may thus be especially important for mental health among BSC who are less concerned about their weight and shape. In other words, the association between experienced weight teasing in childhood and current WBI is stronger for those with low or moderate levels of weight and shape concern, while for those with high weight and shape concern, WBI is generally high, whether or not the BSC reported an experience of weight teasing during childhood. This result, which is implicated in the overall result, could be due to a general negative self-image that is not necessarily weight-related.

Strikingly, the positive association between ES and depressive symptoms was not moderated by weight and shape concern, against the hypothesis. Although the association between ES and depressive symptoms was significant, as expected, it was only weak, which is congruent with the results of a community study of adults with a mean BMI of 36 kg/m^2^ [18], but contrasts previous cross-sectional evidence showing strong correlations between ES and depressive symptoms in adults with BMI between 28 and 45 kg/m^2^ seeking behavioral weight loss treatment [24]. The fact that the strength of association between ES and depressive symptoms was not affected by the level of weight and shape concern suggests that BSC with high weight and shape concern and a high frequency of experienced teasing do not suffer from stronger depressive symptoms than BSC with low weight and shape concern and a low frequency of experienced teasing. Given the large association of weight and shape concern and depressive symptoms, and the small association between ES and depressive symptoms, the result may suggest that weight and shape concern alone are relevant for patients’ mental health, but do not serve as protective or adverse mechanisms between childhood teasing and current depressive symptoms.

The strengths of this study include the large sample size, the multicenter design, and the application of internationally well-established self-report instruments. Among the limitations, data were only quasi-longitudinal with patients reporting their current perceptions of weight teasing in childhood, thus precluding causal interpretations of the present results. Although the WBIS refers to experienced weight-based teasing in childhood, there was no objective information on patients’ weight status in childhood available. With Tylka et al.’s model [14], postulating a rather longitudinal mediation, it must be taken into account that this cross-sectional study’s level of evidence is lower compared to those of longitudinal studies, offering the possibility to establish causality. Further, socially desirable response behavior cannot be completely ruled out, even if the participants were informed that their answers played no role in the clinical decision to have surgery.

## 5. Conclusions

This study cross-sectionally confirms that WBI acts as a mediator between ES and depressive symptoms, and that weight and shape concern have moderating effects on this mediating pathway among BSC. Clinically, the results suggest that the reduction in WBI in BSC may be a valuable target in psychological intervention. The strong correlation between WBI and depressive symptoms shown in this study, high prospective associations between presurgical and postsurgical depressive symptoms [58], and predictive effects of presurgical WBI for diminished weight loss after surgery [59] support pre-surgical assessments as well as improvements in WBI and depressive symptoms as clinical necessities in BSC [60]. A related intervention target could be weight and shape concern. Herein, patients with low weight and shape concern should not be neglected, because for these patients, the mediating effect of WBI on the association between ES and depressive symptoms was especially strong. Regarding psychotherapy in BSC, the results highlight that not the frequency of ES in the past, which cannot be changed in the present, but internal conditions, including WBI and weight and shape concern, are largely related to mental health.

In order to better understand the etiology of BSC’s depressive symptoms and postsurgical outcomes, future research should use longitudinal designs prospectively assessing weight-based teasing in childhood, pre- and postsurgical WBI and outcomes. Due to the importance of WBI for mental health among the population and people with overweight or obesity [61,62,63], intervention studies aiming to reduce the level of WBI will be of high clinical interest. The number of recently developed studies evaluating the efficacy of lowering WBI [64,65] is small. They should be built upon in the future, focusing on the vulnerable group of BSC.

## Figures and Tables

**Table 1:** Sample characteristics.

Baseline Characteristics	*n*	*M*/*n*	*SD*/%	Min.	Max.
Sociodemographic variables					
Age	854	46.8	11.6	18	74
Sex	854				
Women		547	67.2		
Men		280	32.8		
Marital status	832				
Single		154	18.5		
Partnership		160	19.2		
Married		430	51.7		
Divorced		63	7.6		
Widowed		25	3.0		
Educational level	759				
≤10 school years		180	23.7		
>10 school years		579	76.3		
Anthropometrics					
BMI, kg/m^2^	854	48.7	8.0	35.0	97.3
Obesity class 2		100	11.7		
Obesity class 3		754	88.3		
Psychological variables					
POTS, 6–30	854	16.0	7.9	6.0	30.0
WBIS, 1–7	854	4.8	1.3	1.3	7.0
PHQ-D, 0–27	854	7.8	5.2	0.0	26.0
EDE-Q WS, 0–6	854	3.7	1.1	0.0	6.0

Note: BMI = body mass index; POTS = Perception of Teasing Scale; WBIS = Weight Bias Internalization Scale; PHQ-D = Patient Health Questionnaire Depression Scale; EDE-Q WS = Eating Disorder Examination Questionnaire: composite of weight and shape concern.

**Table 2:** Correlations of study variables.

	1	2	3	4	5	6	7
Age	−						
2. Sex	−0.11 **	−					
3. BMI	−0.14 ***	−0.03	−				
4. Education	0.02	0.11 **	−0.11 **	−			
5. POTS	−0.36 ***	0.11 **	0.29 ***	−0.01	−		
6. WBIS	−0.17 ***	0.17 ***	0.09 *	−0.03	0.38 ***	−	
7. PHQ-D	0.01	0.09 **	0.14 ***	−0.05	0.27 ***	0.56 ***	−
8. EDE-Q WS	−0.03	0.19 ***	0.09	0.01	0.33 ***	0.72 ***	0.56 ***

Note: BMI = body mass index (kg/m^2^); POTS = Perception of Teasing Scale; WBIS = Weight Bias Internalization Scale; PHQ-D = Patient Health Questionnaire Depression Scale; EDE-Q WS = Eating Disorder Examination Questionnaire: composite of weight and shape concern. * *p* < 0.05. ** *p* < 0.01. *** *p* < 0.001.

**Table 3:** Effects of simple mediation.

Path	Independent Variable	Dependent Variable	*t*	*p*	Direct Effect [95% CI]	Indirect Effect [95% CI]
a	POTS	WBIS	10.206	<0.001	0.06 [0.05, 0.07]	
b	WBIS	PHQ-D	18.018	<0.001	2.23 [2.01, 2.52]	
c	POTS	PHQ-D	7.931	<0.001	0.19 [0.15, 0.25]	
c’	POTS	PHQ-D	2.841	0.005	0.06 [0.03, 0.12]	0.13 [0.10, 0.16]

Note: POTS = Perception of Teasing Scale; WBIS = Weight Bias Internalization Scale; PHQ-D = Patient Health Questionnaire Depression Scale.

**Table 4:** Effects of moderated mediation.

	WBIS (Mediator)			PHQ-D (Outcome)		
	Effect [95% CI]	t	p	Effect [95% CI]	t	p
**Path a**						
POTS	0.02 [0.01, 0.03]	4.97	<0.001			
EDE-Q WS	0.74 [0.68, 0.79]	26.99	<0.001			
POTS × EDE-Q WS	−0.01 [−0.02, −0.00]	−3.08	0.002			
POTS × EDE-Q WS (−1 SD)	0.03 [0.02, 0.04]	5.37	<0.001			
POTS × EDE-Q WS (mean)	0.02 [0.01, 0.03]	4.97	<0.001			
POTS × EDE-Q WS (+1 SD)	0.01 [−0.00, 0.02]	1.86	0.063			
Age	−0.01 [−0.02, −0.01]	−4.21	<0.001			
Sex	−0.01 [−0.13, 0.13]	−0.18	0.860			
BMI	−0.01 [−0.01, 0.00]	−1.35	0.177			
**Path b**						
WBIS				1.55 [1.29, 1.94]	9.57	<0.001
EDE-Q WS				1.38 [0.98, 1.70]	7.77	<0.001
WBIS × EDE-Q WS				0.49 [0.32, 0.72]	5.01	<0.001
WBIS × EDE-Q WS (−1 SD)				0.99 [0.63, 1.41]	5.17	<0.001
WBIS × EDE-Q WS (mean)				1.55 [1.29, 1.94]	9.57	<0.001
WBIS × EDE-Q WS (+1 SD)				2.11 [1.80, 2.62]	10.45	<0.001
Age				0.05 [0.02, 0.08]	3.75	<0.001
Sex				−0.25 [−0.99, 0.22]	−0.84	0.399
BMI				0.05 [0.01, 0.08]	2.83	0.005
**Path c**’						
POTS				0.03 [−0.00, 0.08]	1.53	0.127
POTS × EDE-Q WS				0.03 [−0.01, 0.06]	1.39	0.164
**Overall moderated mediation**						
POTS × WBIS × EDE-Q WS (−1 SD)				0.03 [0.01, 0.05]		
POTS × WBIS × EDE-Q WS (mean)				0.03 [0.02, 0.05]		
POTS × WBIS × EDE-Q WS (+1 SD)				0.02 [−0.00, 0.04]		

Note. BMI = body mass index; POTS = Perception of Teasing Scale; WBIS = Weight Bias Internalization Scale; PHQ-D = Patient Health Questionnaire Depression Scale; EDE-Q WS = Eating Disorder Examination Questionnaire: composite of weight and shape concern.

## Data Availability

The original contributions presented in the study are included in the article, further inquiries can be directed to the corresponding author.

## References

[1] Ng M., Fleming T., Robinson M., Thomson B., Graetz N., Margono C., Mullany E.C., Biryukov S., Abbafati C., Abera S.F. (2014). Global, regional, and national prevalence of overweight and obesity in children and adults during 1980–2013: A systematic analysis for the Global Burden of Disease Study 2013. Lancet.

[2] The GBD 2015 Obesity Collaborators (2017). Health effects of overweight and obesity in 195 countries over 25 years. N. Engl. J. Med..

[3] NCD Risk Factor Collaboration (NCD-RisC) (2016). Trends in adult body-mass index in 200 countries from 1975 to 2014: A pooled analysis of 1698 population-based measurement studies with 19.2 million participants. Lancet.

[4] World Health Organisation Obesity and Overweight. https://www.who.int/news-room/fact-sheets/detail/obesity-and-overweight.

[5] Chu D.-T., Minh Nguyet N.T., Dinh T.C., Thai Lien N.V., Nguyen K.-H., Nhu Ngoc V.T., Tao Y., Le Son H., Le D.-H., Nga V.B. (2018). An update on physical health and economic consequences of overweight and obesity. Diabetes Metab. Syndr..

[6] Avila C., Holloway A.C., Hahn M.K., Morrison K.M., Restivo M., Anglin R., Taylor V.H. (2015). An overview of links between obesity and mental health. Curr. Obes. Rep..

[7] Lehnert T., Sonntag D., Konnopka A., Riedel-Heller S., König H.-H. (2013). Economic costs of overweight and obesity. Best Practice & Research. Clin. Endocrinol. Metab..

[8] LeBlanc E.S., Patnode C.D., Webber E.M., Redmond N., Rushkin M., O’Connor E.A. (2018). Behavioral and pharmacotherapy weight loss interventions to prevent obesity-related morbidity and mortality in adults: Updated evidence report and systematic review for the US Preventive Services Task Force. JAMA.

[9] Wadden T.A., Bailey T.S., Billings L.K., Davies M., Frias J.P., Koroleva A., Lingvay I., O’Neil P.M., Rubino D.M., Skovgaard D. (2021). Effect of subcutaneous semaglutide vs placebo as an adjunct to intensive behavioral therapy on body weight in adults with overweight or obesity: The STEP 3 randomized clinical trial. JAMA.

[10] Wolfe B.M., Kvach E., Eckel R.H. (2016). Treatment of obesity: Weight loss and bariatric surgery. Circ. Res..

[11] Hilbert A., Staerk C., Strömer A., Mansfeld T., Sander J., Seyfried F., Kaiser S., Dietrich A., Mayr A. (2022). Nonnormative eating behaviors and eating disorders and their associations with weight loss and quality of life during 6 years following obesity surgery. JAMA Netw. Open.

[12] Duarte-Guerra L.S., Coêlho B.M., Santo M.A., Wang Y.-P. (2015). Psychiatric disorders among obese patients seeking bariatric surgery: Results of structured clinical interviews. Obes. Surg..

[13] Fischer L., Wekerle A.-L., Sander J., Nickel F., Billeter A.T., Zech U., Bruckner T., Müller-Stich B.P. (2017). Is there a reason why obese patients choose either conservative treatment or surgery?. Obes. Surg..

[14] Tylka T.L., Annunziato R.A., Burgard D., Daníelsdóttir S., Shuman E., Davis C., Calogero R.M. (2014). The weight-inclusive versus weight-normative approach to health: Evaluating the evidence for prioritizing well-being over weight loss. J. Obes..

[15] Forbes Y., Donovan C. (2019). The role of internalised weight stigma and self-compassion in the psychological well-being of overweight and obese women. Aust. Psychol..

[16] Pötzsch A., Rudolph A., Schmidt R., Hilbert A. (2018). Two sides of weight bias in adolescent binge-eating disorder: Adolescents’ perceptions and maternal attitudes. Int. J. Eat. Disord..

[17] Olson K.L., Mensinger J.L. (2019). Weight-related stigma mediates the relationship between weight status and bodily pain: A conceptual model and call for further research. Body Image.

[18] Hayward L.E., Vartanian L.R., Pinkus R.T. (2018). Weight stigma predicts poorer psychological well-being through internalized weight bias and maladaptive coping responses. Obesity.

[19] Carr D., Friedman M.A. (2005). Is obesity stigmatizing? Body weight, perceived discrimination, and psychological well-being in the United States. J. Health Soc. Behav..

[20] Puhl R.M., Himmelstein M.S., Quinn D.M. (2018). Internalizing weight stigma: Prevalence and sociodemographic considerations in US adults. Obesity.

[21] Braun T.D., Gorin A.A., Puhl R.M., Stone A., Quinn D.M., Ferrand J., Abrantes A.M., Unick J., Tishler D., Papasavas P. (2021). Shame and self-compassion as risk and protective mechanisms of the internalized weight bias and emotional eating link in individuals seeking bariatric surgery. Obes. Surg..

[22] Luck-Sikorski C., Bernard M. (2021). Stigmatisierung und Diskriminierung von Patient*innen mit Adipositas. Psychotherapeut.

[23] Alimoradi Z., Golboni F., Griffiths M.D., Broström A., Lin C.-Y., Pakpour A.H. (2020). Weight-related stigma and psychological distress: A systematic review and meta-analysis. Clin. Nutr..

[24] Crockett K.B., Borgatti A., Tan F., Tang Z., Dutton G. (2022). Weight discrimination experienced prior to enrolling in a behavioral obesity intervention is associated with treatment response among black and white adults in the Southeastern U.S. Int. J. Behav. Med..

[25] Duan W., Wang Z. (2019). Mindfulness capability mediates the association between weight-based stigma and negative emotion symptoms. Mindfulness.

[26] Emmer C., Bosnjak M., Mata J. (2020). The association between weight stigma and mental health: A meta-analysis. Obes. Rev. Off. J. Int. Assoc. Study Obes..

[27] Pearl R.L., Puhl R.M. (2018). Weight bias internalization and health: A systematic review. Obes. Rev. Off. J. Int. Assoc. Study Obes..

[28] Spahlholz J., Pabst A., Riedel-Heller S.G., Luck-Sikorski C. (2016). Coping with perceived weight discrimination: Testing a theoretical model for examining the relationship between perceived weight discrimination and depressive symptoms in a representative sample of individuals with obesity. Int. J. Obes..

[29] Pearl R.L., Hernandez M., Bach C., Groshon L., Wadden T.A. (2023). Prevalence of diagnosed psychiatric disorders among adults who have experienced and internalized weight stigma. Obes. Sci. Pract..

[30] Hoffmann K., Paczkowska A., Bryl W., Marzec K., Raakow J., Pross M., Berghaus R., Nowakowska E., Kus K., Michalak M. (2022). Comparison of perceived weight discrimination between Polish and German patients underwent bariatric surgery or endoscopic method versus conservative treatment for morbid obesity: An International Multicenter Study. Nutrients.

[31] Wagner A.F., Butt M., Rigby A. (2020). Internalized weight bias in patients presenting for bariatric surgery. Eat. Behav..

[32] Pearl R.L., Puhl R.M. (2016). The distinct effects of internalizing weight bias: An experimental study. Body Image.

[33] Szwimer E., Mougharbel F., Goldfield G.S., Alberga A.S. (2020). The association between weight-based teasing from peers and family in childhood and depressive symptoms in childhood and adulthood: A systematic review. Curr. Obes. Rep..

[34] Bidstrup H., Brennan L., Kaufmann L., La Piedad Garcia X.D. (2022). Internalised weight stigma as a mediator of the relationship between experienced/perceived weight stigma and biopsychosocial outcomes: A systematic review. Int. J. Obes..

[35] Magallares A., Bolaños-Rios P., Ruiz-Prieto I., Benito de Valle P., Irles J.A., Jáuregui-Lobera I. (2017). The mediational effect of weight self-stigma in the relationship between blatant and subtle discrimination and depression and anxiety. Span. J. Psychol..

[36] Braun T.D., Quinn D.M., Stone A., Gorin A.A., Ferrand J., Puhl R.M., Sierra J., Tishler D., Papasavas P. (2020). Weight bias, shame, and self-compassion: Risk/protective mechanisms of depression and anxiety in prebariatic surgery patients. Obesity.

[37] Gee A., Troop N.A. (2003). Shame, depressive symptoms and eating, weight and shape concerns in a non-clinical sample. Eat. Weight Disord. EWD.

[38] Oliveira S., Pires C., Ferreira C. (2020). Does Recall Caregiv. Eat. Messages Exacerbate Pathog. Impact Shame Eat. Weight-Relat. Difficulties?. Eat. Weight Disord. EWD.

[39] Troop N.A., Allan S., Serpell L., Treasure J.L. (2008). Shame in women with a history of eating disorders. Eur. Eat. Disord. Rev. J. Eat. Disord. Assoc..

[40] Losekam S., Kraeling S., Goetzky B., Rief W., Hilbert A. (2017). Evaluation of the German version of the Perception of Teasing Scale (POTS). Unveröff. Manuskript. Univ. Marburg.

[41] Thompson J.K., Cattarin J., Fowler B., Fisher E. (1995). The Perception of Teasing Scale (POTS): A revision and extension of the Physical Appearance Related Teasing Scale (PARTS). J. Personal. Assess..

[42] Löwe B., Spitzer R.L., Zipfel S., Herzog W. PHQ_D. Gesundheitsfragebogen für Patienten. Manual. Komplettversion und Kurzform: Autorisierte Deutsche Version des “Prime MD Patient Health Questionnaire (PHQ)”. 2nd ed.; 2002. https://www.klinikum.uni-heidelberg.de/fileadmin/psychosomatische_klinik/download/phq_manual1.pdf.

[43] Löwe B., Spitzer R.L., Zipfel S., Herzog W. (2003). PHQ-D. Gesundheitsfragebogen Für Patienten. Z. Für Med. Psychol..

[44] Spitzer R.L., Kroenke K., Williams J.B. (1999). Validation and utility of a self-report version of PRIME-MD: The PHQ primary care study. Primary care evaluation of mental disorders. Patient Health Questionnaire. JAMA.

[45] American Psychiatric Association (2000). Diagnostic and Statistical Manual of Mental Disorders.

[46] Durso L.E., Latner J.D. (2008). Understanding self-directed stigma: Development of the weight bias internalization scale. Obesity.

[47] Hilbert A., Baldofski S., Zenger M., Löwe B., Kersting A., Braehler E. (2014). Weight bias internalization scale: Psychometric properties and population norms. PLoS ONE.

[48] Pearl R.L., Puhl R.M. (2014). Measuring internalized weight attitudes across body weight categories: Validation of the modified weight bias internalization scale. Body Image.

[49] Schraven S., Hübner C., Eichler J., Mansfeld T., Sander J., Seyfried F., Kaiser S., Dietrich A., Schmidt R., Hilbert A. (2023). Psychometric properties of the WBIS/-M in a representative prebariatric sample–Evidence for a 10-item version. Obes. Facts.

[50] Fairburn C.G., Beglin S.J. (1994). Assessment of eating disorders: Interview or self-report questionnaire?. Int. J. Eat. Disord..

[51] Hilbert A., Tuschen-Caffier B., Karwautz A., Niederhofer H., Munsch S. (2007). Eating Disorder Examination-Questionnaire. Diagnostica.

[52] Hrabosky J.I., Masheb R.M., White M.A., Grilo C.M. (2007). Overvaluation of shape and weight in binge eating disorder. J. Consult. Clin. Psychol..

[53] Grilo C.M., Hrabosky J.I., White M.A., Allison K.C., Stunkard A.J., Masheb R.M. (2008). Overvaluation of shape and weight in binge eating disorder and overweight controls: Refinement of a diagnostic construct. J. Abnorm. Psychol..

[54] Fritz M.S., Mackinnon D.P. (2007). Required sample size to detect the mediated effect. Psychol. Sci..

[55] Hayes A.F. (2022). Introduction to Mediation, Moderation, and Conditional Process Analysis: A Regression-Based Approach (Methodology in the Social Sciences).

[56] Godoy-Izquierdo D., Lara R., Ogallar A., Rodríguez-Tadeo A., Ramírez M.J., Navarrón E., Arbinaga F. (2021). Psychosocial and diet-related lifestyle clusters in overweight and obesity. Int. J. Environ. Res. Public Health.

[57] Lawson J.L., Schuh L.M., Creel D.B., Blackinton R.M., Giambrone S.A., Grilo C.M., Ivezaj V. (2021). Examining weight bias and loss-of-control eating among individuals seeking bariatric surgery. Obes. Surg..

[58] White M.A., Kalarchian M.A., Levine M.D., Masheb R.M., Marcus M.D., Grilo C.M. (2015). Prognostic significance of depressive symptoms on weight loss and psychosocial outcomes following gastric bypass surgery: A prospective 24-month follow-up study. Obes. Surg..

[59] Lent M.R., Napolitano M.A., Wood G.C., Argyropoulos G., Gerhard G.S., Hayes S., Foster G.D., Collins C.A., Still C.D. (2014). Internalized weight bias in weight-loss surgery patients: Psychosocial correlates and weight loss outcomes. Obes. Surg..

[60] Kruseman M., Leimgruber A., Zumbach F., Golay A. (2010). Dietary, weight, and psychological changes among patients with obesity, 8 years after gastric bypass. J. Am. Diet. Assoc..

[61] Curll S.L., Brown P.M. (2020). Weight stigma and psychological distress: A moderated mediation model of social identification and internalised bias. Body Image.

[62] Levy M., Nguyen A., Kakinami L., Alberga A.S. (2021). Weight bias internalization: Relationships with mental health, physical activity, and sedentary behavior. Stigma Health.

[63] Pearl R.L., Puhl R.M., Himmelstein M.S., Pinto A.M., Foster G.D. (2020). Weight stigma and weight-related health: Associations of self-report measures among adults in weight management. Ann. Behav. Med. A Publ. Soc. Behav. Med..

[64] Pearl R.L., Wadden T.A., Bach C., Tronieri J.S., Berkowitz R.I. (2020). Six-month follow-up from a randomized controlled trial of the Weight BIAS Program. Obesity.

[65] Selensky J.C., Carels R.A. (2021). Weight stigma and media: An examination of the effect of advertising campaigns on weight bias, internalized weight bias, self-esteem, body image, and affect. Body Image.

